# β-catenin-mediated hair growth induction effect of 3,4,5-tri-*O*-caffeoylquinic acid

**DOI:** 10.18632/aging.102048

**Published:** 2019-06-29

**Authors:** Meriem Bejaoui, Myra O. Villareal, Hiroko Isoda

**Affiliations:** 1 School of Integrative and Global Majors (SIGMA) University of Tsukuba, Tsukuba City, 305-8572, Japan; 2 Faculty of Life and Environmental Sciences University of Tsukuba, Tsukuba City, 305-8572, Japan; 3 Alliance for Research on the Mediterranean and North Africa (ARENA) University of Tsukuba, Tsukuba City, 305-8572, Japan

**Keywords:** 3,4,5-tri-*O*-caffeoylquinic acid (TCQA), β-catenin, dermal papilla, anagen, Wnt/ β-catenin pathway

## Abstract

The hair follicle is a complex structure that goes through a cyclic period of growth (anagen), regression (catagen), and rest (telogen) under the regulation of several signaling pathways, including Wnt/ β-catenin, FGF, Shh, and Notch. The Wnt/β-catenin signaling is specifically involved in hair follicle morphogenesis, regeneration, and growth. β-catenin is expressed in the dermal papilla and promotes anagen induction and duration, as well as keratinocyte regulation and differentiation. In this study, we demonstrated the activation of β-catenin by a polyphenolic compound 3,4,5-tri-*O*-caffeoylquinic acid (TCQA) in mice model and in human dermal papilla cells to promote hair growth cycle. A complete regrowth of the shaved area of C3H mice was observed upon treatment with TCQA. Global gene expression analysis using microarray showed an upregulation in hair growth-associated genes. Moreover, the expression of β-catenin was remarkably upregulated *in vivo* and *in vitro*. These findings suggest that β-catenin activation by TCQA promoted the initiation of the anagen phase of the hair cycle.

## Introduction

Hair plays an important role in an individual's general appearance [1]. Hair loss is a common disorder that occurs both in men and women. Although it is not dangerous or severe, it affects people’s quality of life and leads to psychological changes that lower self-esteem and even disturbing an individual capability to fulfill a normal lifestyle [1–3].

The hair follicle (HF) is composed of two compartments, the epidermal (epithelial) and dermal (mesenchymal), and is formed via a coordinated and complicated crosstalk between dermal cells playing the role of inducers and epithelial cells as responders. The mesenchymal is composed of specialized fibroblasts divided into the dermal papilla (DP), located at the proximal end of the HF and surrounded by matrix cells, and the dermal sheath (DS), considered as a reservoir of DP cells [4]. The DP plays an important role in the induction and maintenance of the hair growth cycle by acting as inductive signal while driving the differentiation of epithelial stem cells residing in the bulge area of the HF, and generating the complex follicular product, the shaft, and the sheath [5–8]. In the adult hair, the HF undergo continuous self-renewal and cycling, dividing in three phases anagen, catagen, and telogen [3]. During the growth phase or anagen, the DP regulates the migration of bulge residing stem cells to the bulb region forming the matrix cells that will be proliferating and dividing into the hair shaft and the inner root sheath (IRS) [9,10]. The HF enters then a regression phase known as catagen where the hair matrix cells stop proliferating and the hair is attached to a keratin matrix above the DP [11]. During the third phase (telogen), the follicle is resting and the hair shaft is dislodged in non-active follicle. In order to initiate the transition from telogen to anagen, various signaling pathways play a key role in cell proliferation and differentiation during development and homeostasis of the HF [12]. Previous studies have identified Wnt, FGF, Shh, and Notch signaling and several transcription factors as regulators of the hair cycle. Wnt/β-catenin signaling pathway plays a major role in the development, growth, and proliferation of the HF and the regulation of the activity of embryonic and adult stem cell populations [13,14]. The activation of Wnt/β-catenin is initiated by Wnt ligand that binds to frizzled (FZD) and LRP5/6 co-receptors to form a complex inducing β-catenin activation and translocation to the nucleus. Therefore, β-catenin activates canonical target genes involved in the growth of the HF including, Wnt signaling proteins expressed during anagen, and FGF signaling molecules involved in keratinocytes differentiation [15–17]. Wnt/β-catenin activation in the HF initiates the anagen induction by inducing the proliferation and differentiation of the epithelial matrix cells that produces the hair shaft and IRS [18].

Studies have shown that the lack of β-catenin inhibit the initiation of hair growth cycle and its activation in the DP promotes postnatal hair growth and the elongation of anagen phase [19–21]. Activating β-catenin expression is therefore considered significant in the initiation of the anagen phase and promotion of hair growth cycle. Recently, drugs like minoxidil and finasteride are used to promote hair growth cycle but they showed side effects [22–24].

In this context, developing a drug with no undesirable side effects is becoming urgent in order to have an alternative therapy for promoting hair growth. Caffeoylquinic acid (CQA) is a phenylpropanoids compound exhibiting several beneficial properties including anti-oxidant, anti-allergic, neuroprotective, and melanogenesis-regulating effects [25–28]. TCQA or 3,4,5-tri-*O*-caffeoylquinic acid with IUPAC name (3R,5R)-3,4,5-tris[[(E)-3-(3,4-dihydroxyphenyl)prop-2-enoyl]oxy]-1-hydroxycyclohexane-1-carboxylic acid, is a CQA derivative and chlorogenic acid (CGA) family member compound, that has a stable albumin affinity and is composed of multi-esters formed between quinic acid and one-to-four residues of trans-cinnamic acids . TCQA has been found to induce a powerful inhibitory activities against aldose reductase, hypertension, hyperglycemia, and Alzheimer's disease without unwanted secondary effects [29]. Moreover, TCQA induces neurogenesis, improves learning and memory in aged mice, and promotes the differentiation of human neural stem cells [30], however its effect on Wnt/β-catenin signaling pathway and hair growth promotion has not yet been assessed. This study was designed to elucidate the ability of TCQA to activate β-catenin and its target genes to promote hair growth, anagen induction and elongation in 8-weeks-old C3H male mice. Global gene expression analysis was performed to explain the hair growth-promoting effects. Furthermore, β-catenin expression in DP and its effect on DP cell proliferation was evaluated.

## RESULTS

### 3,4,5-tri-*O*-caffeoylquinic acid (TCQA) promoted hair regrowth in C3H mice

**Figure 1:**
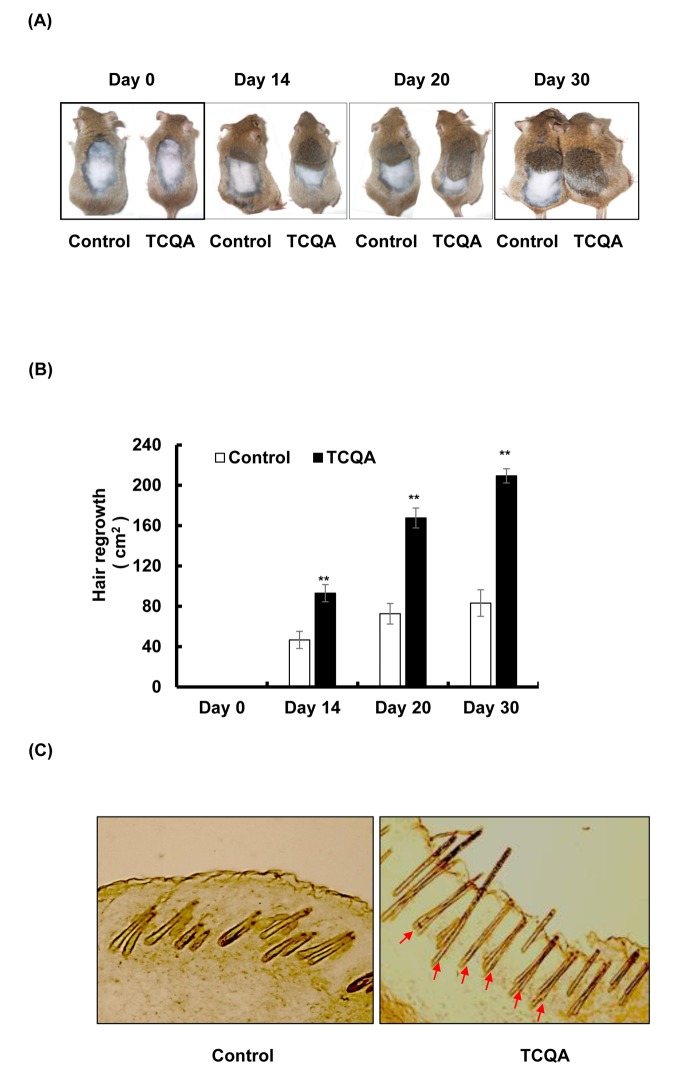
**TCQA promoted hair regrowth in C3H mice skin.** (**A**) The back skin of eight-weeks-old male C3H mice was shaved and treated daily with topical application of 1 wt% TCQA (1 g TCQA in 100 ml milli-Q water) and with milli-Q water (control) for 30 days. (**B**) The area of the new generated coat was measured by ImageJ. (**C**) Skin from treated area from TCQA-treated group and control group were cut at thickness of 10 µM and visualized under the microscope. *Statistically significant (*P* ≤0.05) difference between control and TCQA-treated group. **Statistically significant (*P* ≤0.01) difference between control and TCQA-treated mice.

The ability of TCQA to promote hair regrowth was tested in eight-weeks-old male C3H mice. Ten days after shaving, TCQA-treated group exhibited hair regrowth in the shaved area (Figure 1A). TCQA significantly enhanced the hair growth by approximately 40%, 80%, and 120% at day 14, 20, and 30, respectively, compared with the control (Figure 1B). By the end of the treatment period (day 30), the treated group displayed a markedly complete regrowth of the hair in the clipped area. In contrast, for the control group, only 37% hair regrowth area was observed (Figure 1A and 1B). The direction of the growth was random in the treated and untreated mice. These results showed that TCQA stimulated the hair growth cycle. Minoxidil, a widely used drug against alopecia often used as a positive control ,induces hair regrowth in C3H mice within 28 days [31]. Our results showed that TCQA had the same effect as minoxidil in stimulating hair growth cycle. Interestingly, the hair shaft was fully developed and the epidermis and the hair follicle (HF) displayed no sign of inflammation or irritation following TCQA application (Figure 1C). The anagen phase is divided into six sub-stages that goes from I to VI and starting the third phase the HFs development can be distinguished. As shown in Figure 1C (indicated by arrows), the HF from the TCQA-treated mice were at advanced stage of anagen phase. The results indicated that TCQA induced the transition of the hair cycle from telogen to anagen phase accelerating hair growth *in vivo*.

### Gene expression changes in C3H mice skin caused by 3,4,5-tri-*O*-caffeoylquinic acid (TCQA)

Microarray analysis was conducted from skin collected from the treated area with TCQA and milli-Q water to elucidate the mechanism behind the observed hair growth promoting effect of TCQA. The expression of 1235 genes was modulated by TCQA out of which, 435 were upregulated while 800 downregulated. Genes relevant in β-catenin binding, epidermal growth factor, transcription regulation, and Wnt signaling were upregulated (Figure 2A). On the other hand, genes involved in glycosylation, protein binding, and β-catenin degradation complex were downregulated (Figure 2B). A volcano plot was created to compare the gene expression in the control group vs TCQA group (Figure 2C). TCQA significantly upregulated 435 genes (2-fold change; red) and downregulated 800 genes (2-fold change; green), while the gray color represents the other genes (≤2-fold change) (Figure 2C).

**Figure 2AB:**
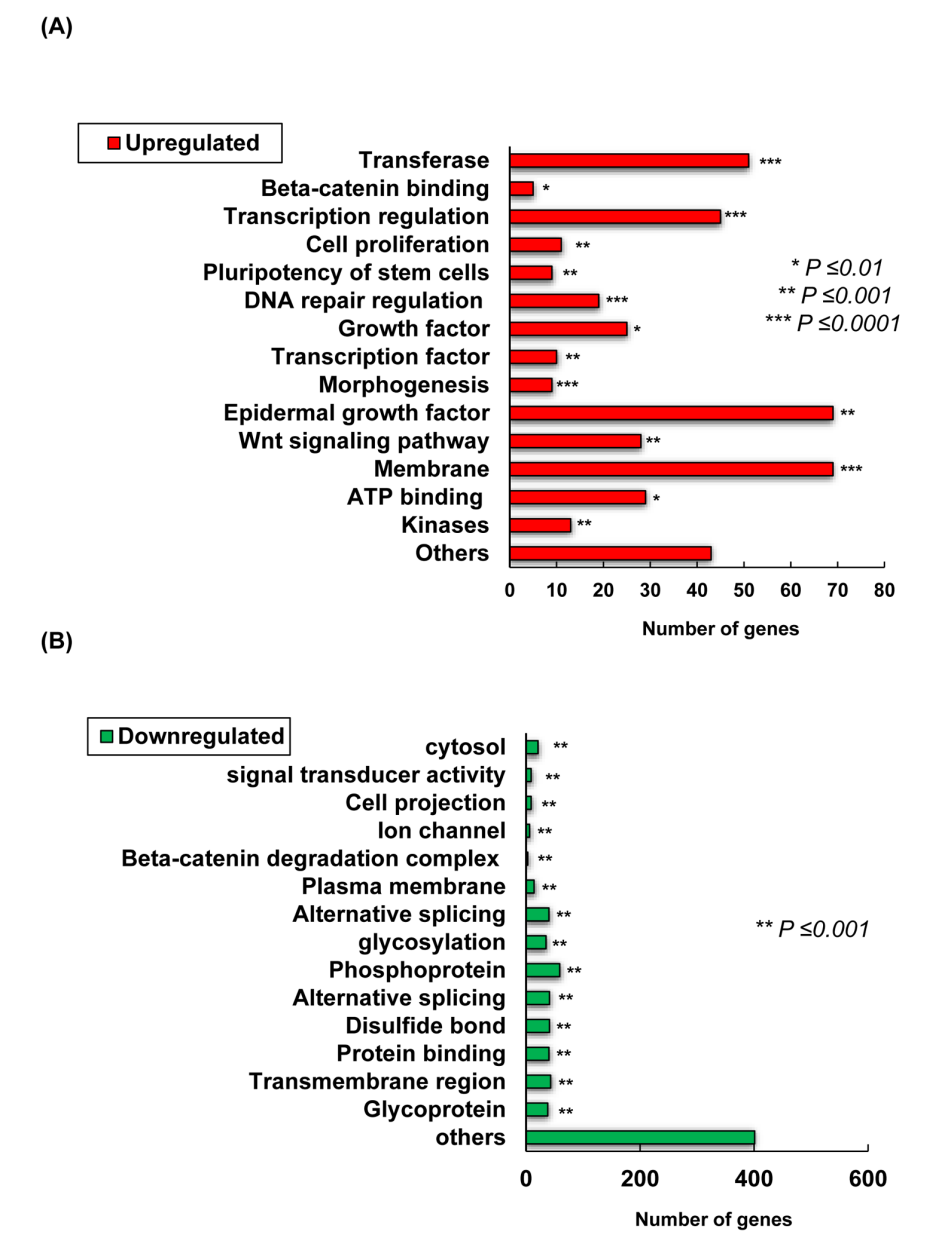
** Transcriptome changes induced by TCQA, 1235 genes were significantly selected: 435 were upregulated and 800 downregulated.** (**A**) Summary of the functional categories of upregulated genes in response to TCQA treatment. (**B**) Summary of the functional categories of downregulated genes in response to TCQA treatment. Analyses for the down and upregulated genes were performed individually using Database for Annotation, Visualization and Integrated Discovery v6.8 (DAVID). Bars represent the number of genes implicated in each category. *Statistically significant (*P* ≤0.01). **Statistically significant (*P* ≤0.001). ***Statistically significant (*P* ≤0.0001).

**Figure 2CD:**
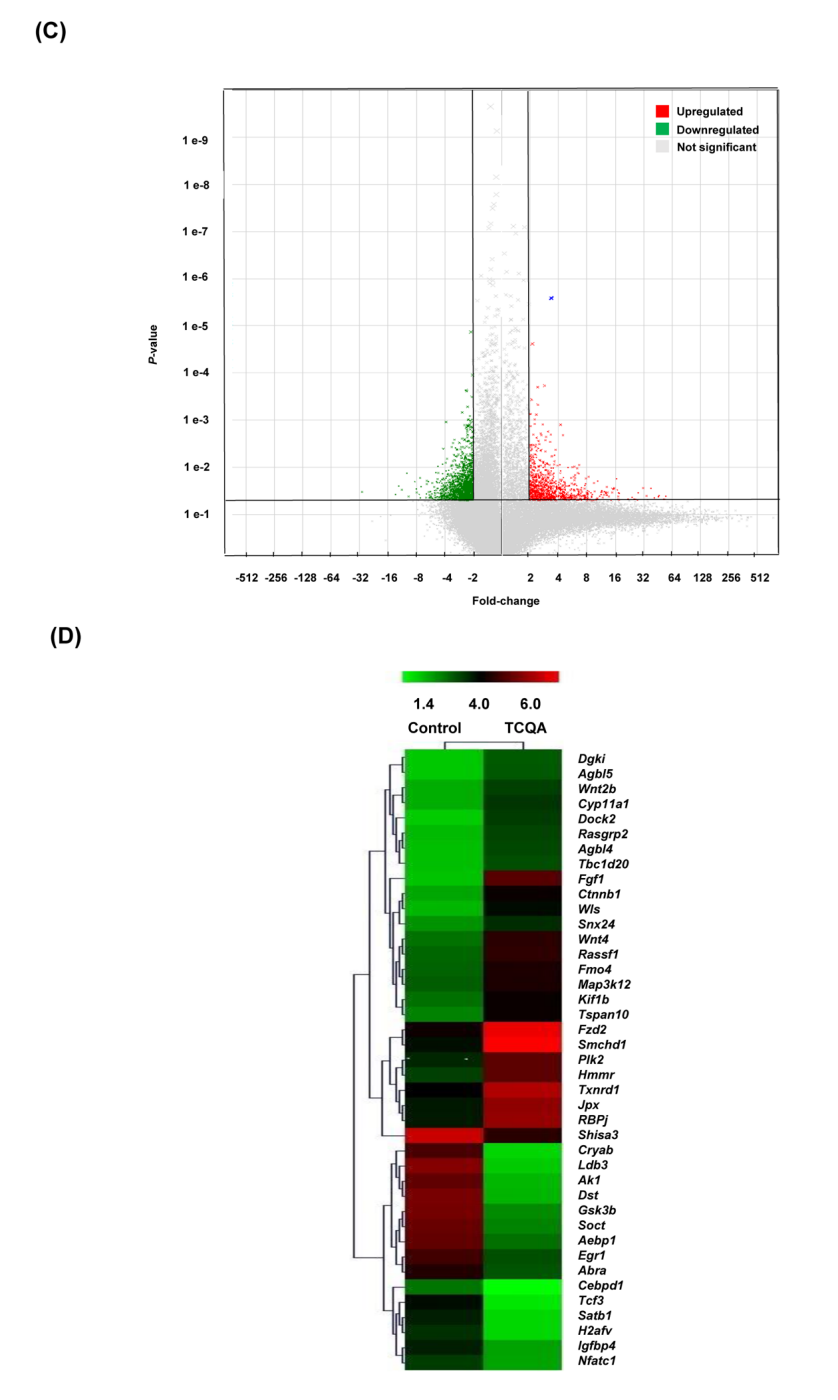
** Transcriptome changes induced by TCQA, 1235 genes were significantly selected: 435 were upregulated and 800 downregulated.** (**C**) Hierarchical clustering of the genes altered after treatment with TCQA using Euclidean distance and average linkage algorithm of the TIGR Mev version 3.0.3 software (The Institute for Genomic Research, MD, USA). Horizontal stripes represent genes and columns represent control and TCQA. The significant fold change in gene expression is 2-fold change (control vs TCQA). (**D**) The volcano plot represents the regulated genes between the control and TCQA. The red color represents the upregulated genes, the green color the downregulated genes, and the grey color the unregulated genes. The expression of the genes above or below, left or right, the lines differed more than 2-fold change between the control and TCQA group.

Genes with 2-fold change in expression (control vs TCQA) were subjected to hierarchical clustering that generated five clusters. In the first cluster (enrichment Score: 1.53), TCQA regulated genes including *Wnt2b, Dock2, Rasgrp2, Agbl4,* and *Dgki* that are relevant for protein binding *(P*=0.0048), Ras signaling pathway (*P*=0.023), and intracellular signal transduction (*P*=0.017), and microtubule *(P*=0.0018) (Figure 2D). Moreover, a regulation of genes including, *Ctnnb1, Wls, Rassf1*, and *Map3k12* that plays a role in ATP binding (*P*=0.023), kinase activity (*P*=0.0152), Wnt signaling (*P*=0.02152), and phosphorylation (*P*=0.025) was observed in cluster two (enrichment score: 0.83). The third cluster with enrichment score of 0.29 is composed of genes that are significant for cell adhesion (*P*=0.0049), cAMP signaling pathway (*P*=0.02), and neuroactive ligand-receptor interaction (*P*=0.0049). The last two clusters with an enrichment score of 0.89 and 0.55, respectively, represent the genes that were downregulated upon treatment with TCQA. Those genes are significant for metal binding (*P*=0.0045), Wnt signaling repression (*P*=0.0015), and cell surface receptor linked signal transduction (*P*=0.0175) (Figure 2D).

Tables 1, 2, 3, and 4 summarize the significantly modulated genes and their biological activities. An upregulation in the expression of canonical Wnt-associated genes, *Ctnnb1, Wls,* *Wnt2b,* and *Wnt4* was observed (Table 1)*.* Notch, FGF, and Rac/Ras pathway-related genes were upregulated as well. Genes significant for keratinocytes differentiation, including *Foxn1, Rps3* were upregulated. In addition, the expression of genes involved in cell differentiation, cell cycle, ATP binding, and oxidation-reduction process like *Sapcd2, Smchd1,* and *Txnrd1,* were enhanced by TCQA (Table 2**)**. Genes associated with telogen phase, repression of Wnt signaling, β-catenin degradation, and aging (*Ldb3, Ak1, Gsk3b, Tcf3, Soct,* and *Nfatc1)*, were downregulated (Tables 3 and 4).

**Table 1:** Top upregulated anagen-associated genes in TCQA-treated mice (vs control) *.

**Gene symbol**	**Gene name**	**Biological function**	**Fold-change**	*P* value **
*Wls*	Wntless	Wnt secretion	3.39	0.026
*Fgf1*	Fibroblast growth factor 1	Hair morphogenesis	3.22	0.026
*Ctnnb1*	Catenin (cadherin associated protein), beta 1	Cell differentiation; hair follicle morphogenesis; hair cycle process; positive regulation of fibroblast growth factor	3.2	0.043
*Wnt4*	Wingless-related MMTV integration site 4	Hair morphogenesis	2.87	0.047
*Tspan10*	Tetraspanin 10	Notch signaling promotion	2.86	0.011
*RBPj*	Recombination signal binding protein for immunoglobulin kappa J region-like	Hair fate determination of hair follicular stem cells	2.72	0.003
*AlpL*	Alkaline phosphatase	Dermal papilla marker	2.33	0.007
*Dlx*	Distal-less homeobox	Regulator of hair follicle differentiation and cycling	2.31	0.034
*Rps3*	Ribosomal protein S3	Positive regulation of NF-kB required for anagen maintenance	2.17	0.000017
*Wnt2b*	Wingless related MMTV integration site 2b	Hair morphogenesis	2.16	0.0002
*Foxn1*	Forkhead box N1	Keratinocyte differentiation; hair follicle development	2.06	0.00015
*Krt14*	Keratin 14	Formation of epithelial hair buds; hair cycle	2.04	0.002
*Corin*	Corin serine peptidase	Dermal papilla marker upregulated during anagen	2.03	0.006
*Fgf2*	Fibroblast growth factor 2	Hair follicle growth	2.02	0.006

*Genes functions were obtained from Mouse Genome Informatics (MGI).

**ANOVA was performed to assess the level of significance between groups. The gene expression was considered significant when fold change was ≥ 2-fold (control vs TCQA).

**Table 2:** Top upregulated genes associated with the observed hair growth in TCQA-treated mice (vs control) *.

**Gene symbol**	**Gene name**	**Biological function**	**Fold- change**	*P* value **
*Smchd1*	SMC hinge domain containing 1	ATP binding	6.83	0.021
*Dock2*	Dedicator of cyto-kinesis 2	Positive regulation of Rac protein signal transduction	2.78	0.00014
*Rassf1*	Ras association (RalGDS/AF-6) domain family member 1	Ras protein signal transduction ; cell cycle	2.69	0.007
*Sapcd2*	Suppressor APC domain containing 2	Cell proliferation; cell cycle	2.66	0.005
*Txnrd1*	Thioredoxin reductase 1	Oxidation-reduction process; protection against oxidative stress	2.63	0.001
*Tada3*	Transcriptional adaptor 3	Stabilization and activation of the p53	2.58	0.011
*Fmo4*	Flavin containing monooxygenase 4	Oxidation-reduction process	2.49	0.0003
*Cyp11a1*	Cytochrome P450, family 11, subfamily a, polypeptide 1	Oxidation-reduction process	2.42	0.003
*Kif1b*	Kinesin family member 1B	ATP binding	2.3	0.004
*Rasgrp2*	RAS, guanyl releasing protein 2	Ras activation	2.28	0.0004
*Agbl4*	ATP/GTP binding protein-like 4	ATP binding	2.2	0.0001
*Dgki*	Diacylglycerol kinase, iota	Ras protein signal transduction regulation	2.17	1.26E-07
*Plk2*	Polo-like kinase 2	Ras protein signal transduction	2.17	0.027
*Map3k12*	Mitogen-activated protein kinase kinase kinase 12	ATP binding, cell-cycle progression	2.11	0.004

*Genes functions were obtained from Mouse Genome Informatics (MGI).

**ANOVA was performed to assess the level of significance between groups. The gene expression was considered significant when fold change was ≥ 2-fold (control vs TCQA)

**Table 3:** Top downregulated genes in TCQA-treated mice (vs control)*.

**Gene symbol**	**Gene name**	**Biological function**	**Fold-change**	*P* value **
*Ldb3*	LIM domain binding 3	Regulator of transcription during telogen	-8.65	0.040
*Cryab*	Crystallin, alpha B	Negative regulator of apoptosis	-6.89	0.035
*Ak1*	Adenylate kinase 1	Cell cycle arrest	-6.24	0.027
*Dst*	Dystonin	Cell cycle arrest	-5.34	0.027
*Gsk3b*	Glycogen synthase kinase 3 beta	Phosphorylation of β-catenin	-5.08	0.002
*Tcf3*	Transcription factor 3	Wnt/β-Catenin repression	-4.72	0.042
*Soct*	Sclerostin	Negative regulation of Wnt/β-Catenin	-4.53	0.013
*Aebp1*	AE binding protein 1	Regulator of telogen hair follicle	-3.8	0.015
*Satb1*	Special AT-rich sequence binding protein 1	Regulator of transcription during telogen	-3.36	0.026
*H2afv*	H2A histone family	Stem cell quiescence	-3.31	0.040
*Egr1*	Early growth response 1	Negative regulation of Wnt/β-Catenin; upregulated with aging	-2.59	0.045
*Igfbp4*	Insulin-like growth factor binding protein 3	Negative regulation of Wnt/β-Catenin	-2.55	0.031
*lL-6*	Interleukin 6	Inflammation response	-2.44	0.026
*Shisa3*	Shisa family member 3	Negative regulator of Wnt/β-Catenin	-2.39	0.002
*Abra*	Actin-binding Rho activating protein	Regulator of transcription during telogen	-2.22	0.046
*Nfatc1*	Nuclear factor of activated T cells, cytoplasmic, calcineurin dependent 1	Stem cells quiescence	-2.09	0.011

*Genes functions were obtained from Mouse Genome Informatics (MGI).

**ANOVA was performed to assess the level of significance between groups. The gene expression was considered significant when fold change was ≥ 2-fold (control vs TCQA)

**Table 4:** Telogen-associated genes downregulated by TCQA*.

**Function**	**Gene symbol**
Cell cycle arrest	*Ak1,Dst*
Regulation of transcription	*Ldb3*,*Abra, Satb, Cebpd1*,*Synpo2, Aebp1, Rora,* *lrrfip*
Negative regulator of cell growth	*Igfbp4*
Anti-apoptosis	*Cryab,Tsc22d3*
Transport	*Rpb7*
Signal transduction	*Hspb6, Obscn*
Carbohydrate metabolic process	*Pygm, Gpd1, Agl*
Proteolysis	*Pcolce*
Aging	*Jun, Egr1, Fos*

*Genes functions were obtained from Mouse Genome Informatics (MGI).

### 3,4,5-tri-*O*-caffeoylquinic acid (TCQA) stimulated β-catenin expression *in vivo*

TCQA-treated mice were observed to have an accelerated anagen phase and at the same time *Ctnnb1* gene expression was observed to be upregulated based on microarray analyses results, the effect of TCQA on β-catenin expression was further determined in mice skin tissue. Results revealed that β-catenin expression in TCQA-treated mice skin was increased in the HF, in the area where the dermal papilla (DP) cells are, in the root sheath, and in the bulb area (Figure 3A). In case of the control mice, β-catenin expression was located in the epidermis and the upper part of the dermis (Figure 3A). In addition, the gene expression of *Ctnnb1* in treated skin tissues was enhanced up to 2.3-fold compared with the control (Figure 3B). This upregulation of *Ctnnb1* expression was followed by an increase in β-catenin protein expression level as shown in Figures 3C and 3D.

**Figure 3AB:**
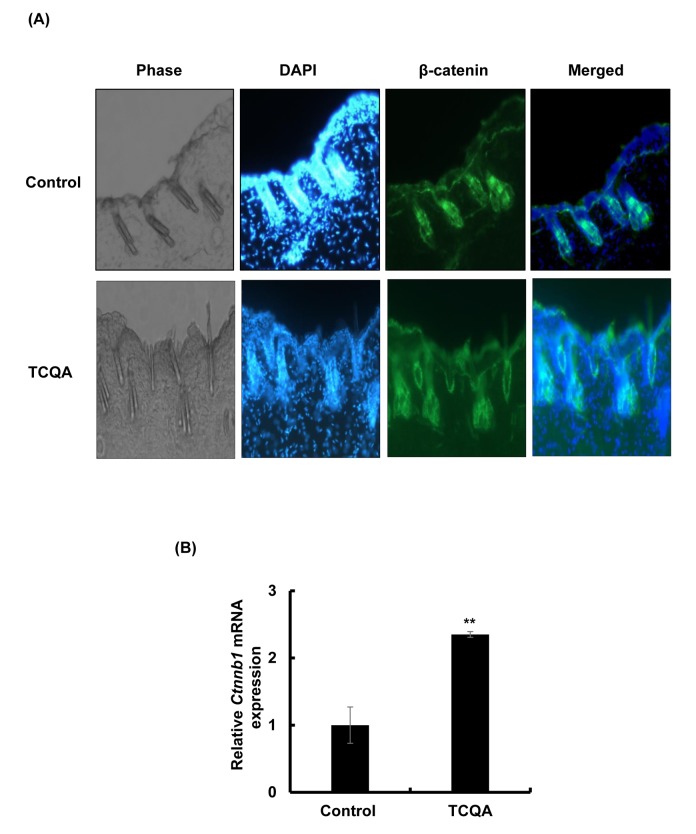
**TCQA enhanced β-catenin expression in the hair follicle.** (**A**) Immunohistochemistry was performed to measure β-catenin expression in the hair follicle and the epidermis in skin collected from the treated area from mice dorsal skin at 30 days after treatment. The figure is divided into four panels, the first panel is the phase, the second is DAPI to stain the nucleus, the third is for β-catenin staining, and the last panel is a merge between β-catenin and the nucleus. (**B**) *Ctnnb1* mRNA relative expression was measured after treatment with TCQA at 30 days after treatment. The mRNA level was quantified using TaqMan real-time PCR from RNA extracted from the treated area (TCQA or milli-Q water) from the mice dorsal back.

**Figure 3C-E:**
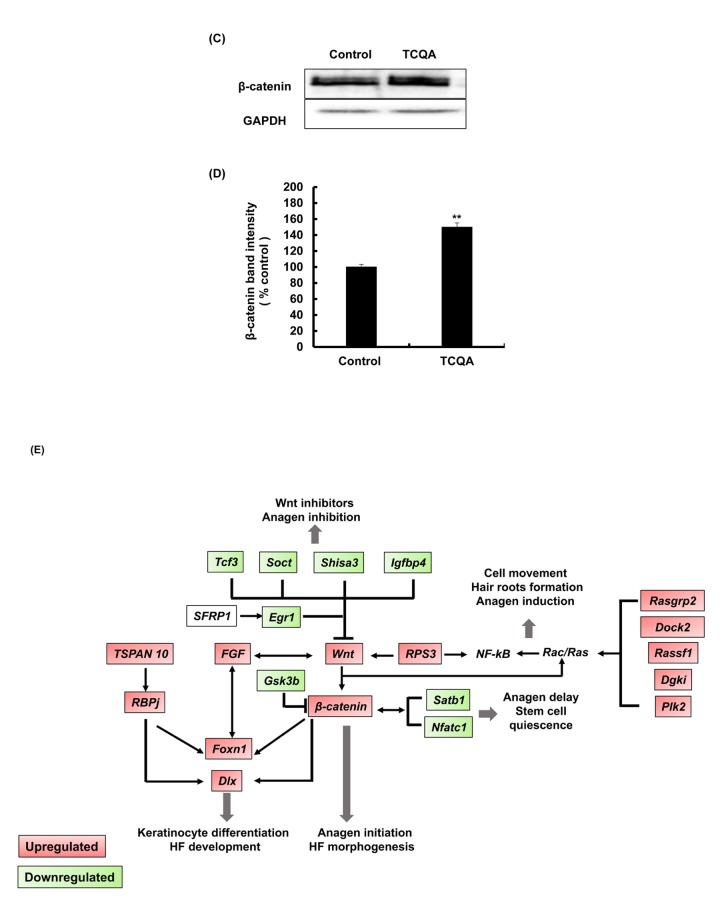
**TCQA enhanced β-catenin expression in the hair follicle.** (**C**) β-catenin protein expression was determined at the end of the treatment period. The protein was extracted from the treated area from the mice dorsal part, and western blot was carried away. (**D**) Band intensities was done assessed using LI-COR system. Results represent the mean ± SD of three independent experiments. *Statistically significant (*P* ≤0.05) difference between control and TCQA-treated mice. **Statistically significant (*P* ≤0.01) difference between control and TCQA-treated mice. (**E**) Summary of the up and downregulated genes modulated by TCQA compared with the control. The red color represents the upregulated genes and the green color the downregulated genes.

Figure 3E illustrates the summary of the modulated genes by TCQA. β-catenin target genes that are involved in HF development and keratinocyte differentiation including, *Foxn1, Dlx, Fgf,* and others, were upregulated. In contrast, genes that inhibit Wnt/β-catenin signaling including *Gsk3b,* *Tcf3,* and *Igfbp4* were downregulated (Figure 3E). These results indicated that the observed hair regrowth effect appears to be caused by increased β-catenin expression following TCQA treatment.

### 3,4,5-tri-*O*-caffeoylquinic acid (TCQA) increased the proliferation of hair bulb cells

**Figure 4:**
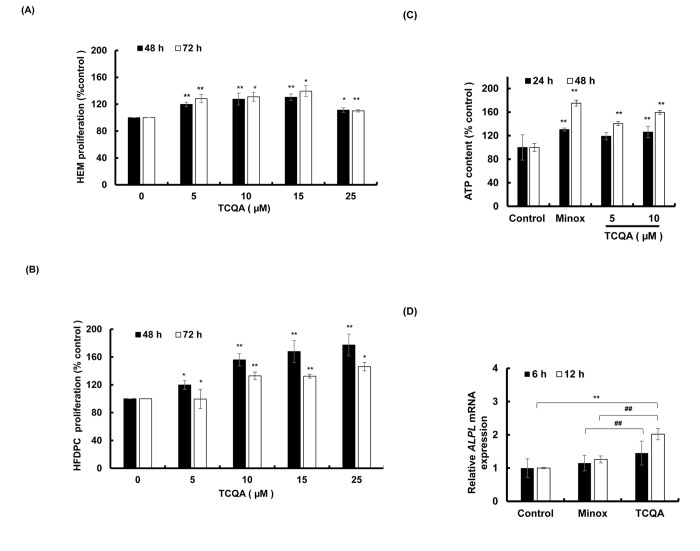
**TCQA stimulated hair bulb cells proliferation.** (**A**) Cell proliferation of human epidermal melanocytes (HEM) was assessed after 48 and 72 h treatment with various concentrations of TCQA. **(B**) Cell proliferation of human hair follicle dermal papilla cells (HFDPCs) was assessed after 48 and 72 h treatment with various concentrations of TCQA. (**C**) ATP content determination after treatment with 5 and 10 µM of TCQA and 0.1 µM of minoxidil (Minox) used as positive control. (**D**) Gene expression of *ALPL* (Alkaline Phosphatase) after 6 and 12 h treatment with 0, 10 µM TCQA, and 0.1 Minox. The mRNA level was quantified using TaqMan real-time PCR after treatment. Results represent the mean ± SD of three independent experiments. *Statistically significant (P ≤0.05) difference between control and treated cells. **Statistically significant (P ≤0.01) difference between control and treated cells. ##Statistically significant (P ≤0.01) difference between Minox-treated cells and TCQA-treated cells.

To determine the cytotoxic effect of TCQA, if any, on the cells that reside in the bulb area of the HF, a proliferation assay (MTT assay) of human epidermal melanocytes (HEM) and human hair follicle dermal papilla cells (HFDPC) was conducted. The cells were treated with 0, 5, 10, 15, and 25 µM TCQA for 48 and 72 h. Results showed that TCQA was not cytotoxic to the cells at all concentrations and in fact, the proliferation of the cells was stimulated (Figure 4A and 4B).

### 3,4,5-tri-*O*-caffeoylquinic acid (TCQA) increased the ATP content of human hair follicle dermal papilla cells (HFDPCs)

The number of hair matrix and the size of the HF is determined by DP cells. 10 µM of TCQA significantly enhanced the ATP content of DP cells by around 60% after 48 h treatment (Figure 4C). This result was supported by the microarray analyses that showed an upregulation in genes related to ATP binding.

### 3,4,5-tri-*O*-caffeoylquinic acid (TCQA) induced alkaline phosphatase (*ALPL)* and β-catenin expression in human hair follicle dermal papilla cells (HFDPCs)

Alkaline phosphatase (ALP) is known to be a marker of DP cells. As microarray results showed an upregulation in its gene expression, the validation using DP cells was carried out. Results showed an upregulation in *ALPL* expression after 6 and 12 h treatment (Figure 4D) confirming that TCQA stimulated the proliferation of DP cells.

The absence of β-catenin in DP cells results to an early entry to catagen phase [32]. To confirm the effect of TCQA on β-catenin stimulation, its protein and gene expression levels in HFDPCs were evaluated. β-catenin was observed to be stimulated by 10 µM TCQA after 12 h treatment, and this expression level was maintained until after 24 h (Figure 5A and 5B). Moreover, according to the immunocytochemistry results, TCQA caused the accumulation and nuclear translocation of β-catenin (Figure 5C). The expression of β-catenin gene (*CTNNB1)* was significantly upregulated after 6 and 12 h treatment with 10 µM TCQA (Figure 5D) suggesting that TCQA promoted hair growth by activating β-catenin expression in the DP at the onset or early stage of anagen phase.

**Figure 5AB:**
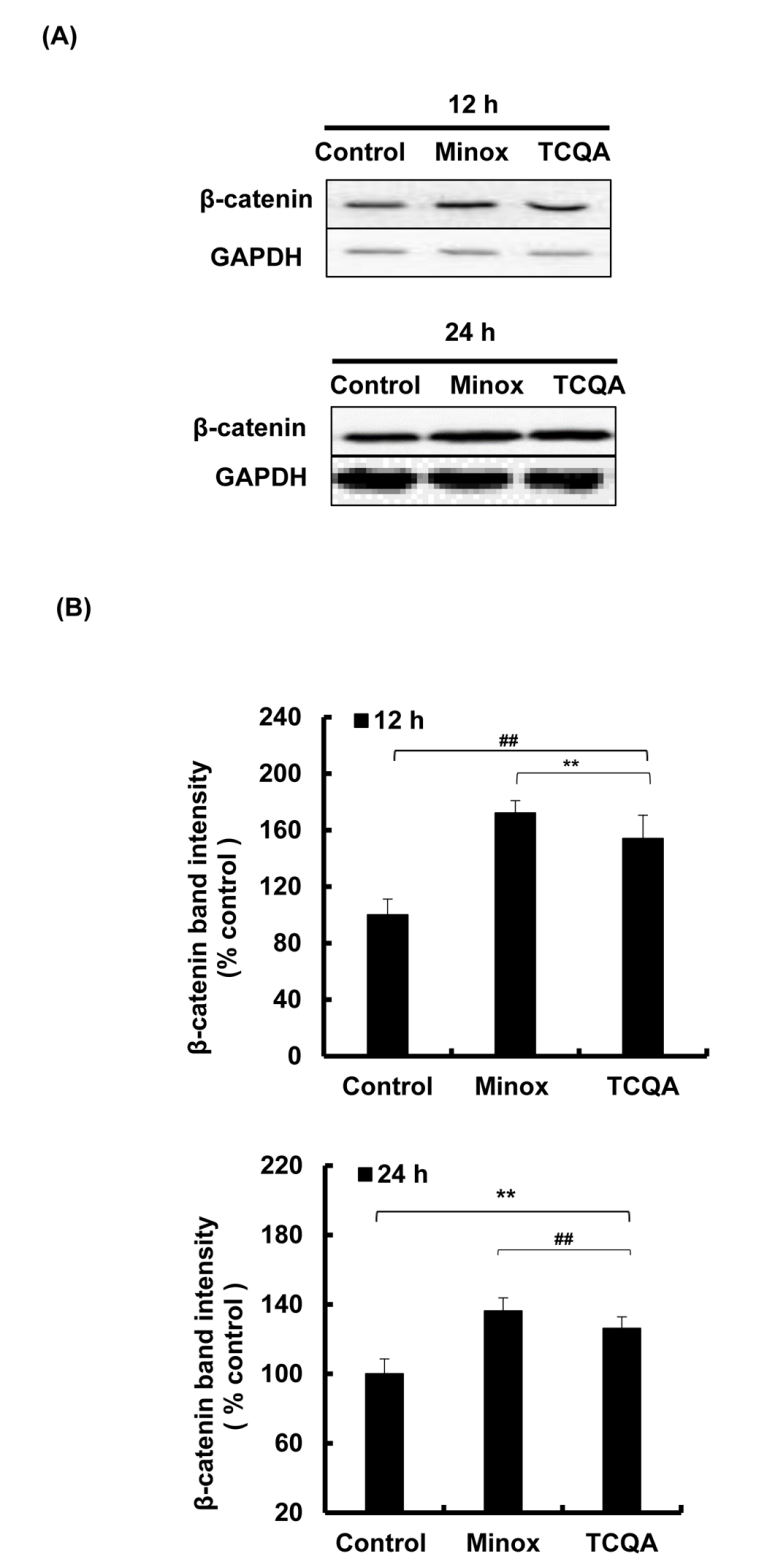
**TCQA stimulated β-catenin expression in human hair follicle dermal papilla cells (HFDPCs).** (**A**) β-catenin protein expression after 12 and 24 h treatment with 0 and 10 µM TCQA and 0.1 µM Minox. (**B**) Band intensities was done using LI-COR system after 12 h and 24 h treatment.

**Figure 5CD:**
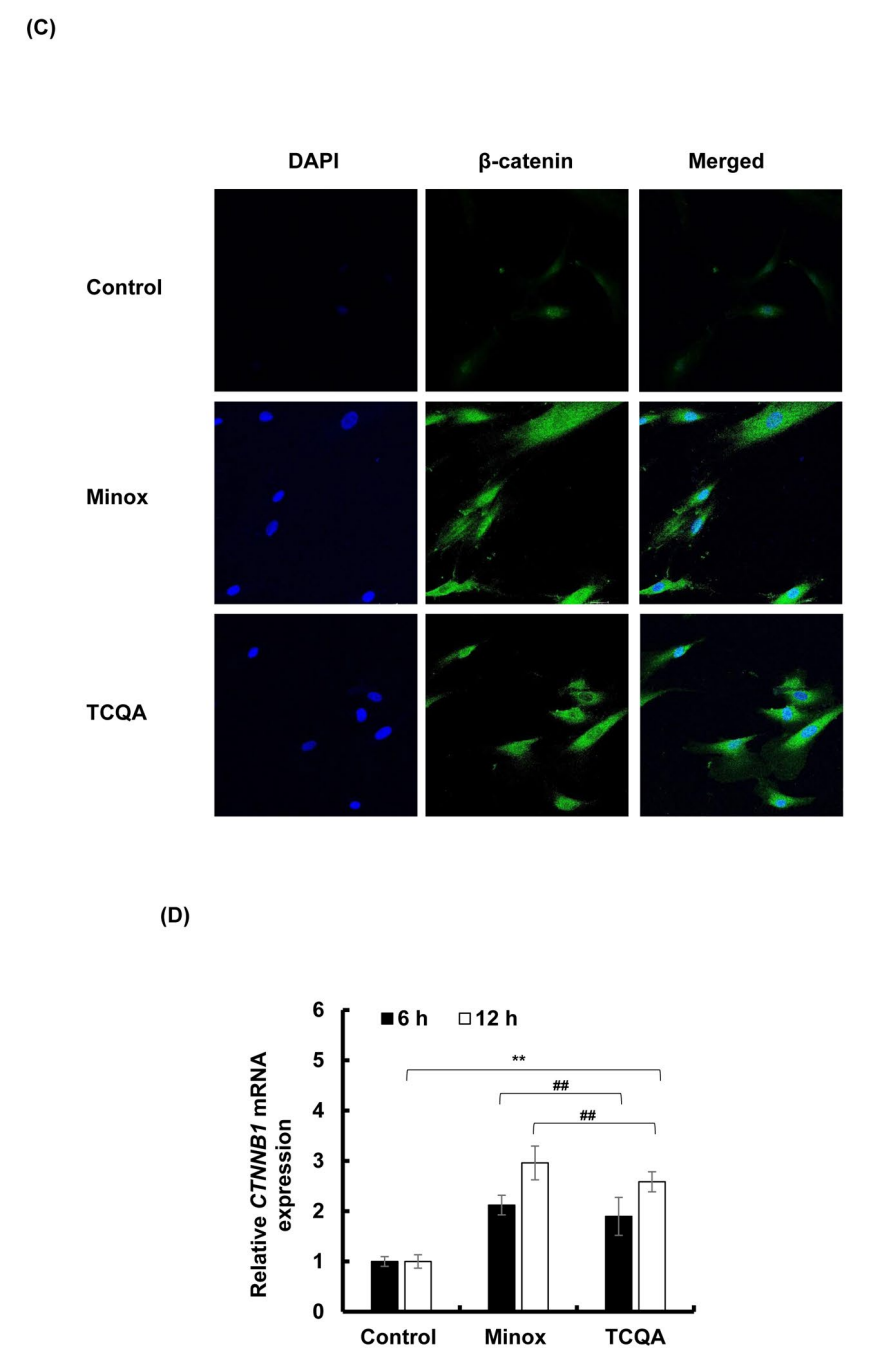
**TCQA stimulated β-catenin expression in human hair follicle dermal papilla cells (HFDPCs).** (**C**) Immunocytochemistry of β-catenin expression in HFDPC after 24 h treatment with 0, 10 µM TCQA and 0.1 µM Minox. Scale bar=25 µm; magnificence 40 X. (**D**) Gene expression of *CTNNB1* (β-catenin) after treatment with 0 and 10 µM TCQA, and 0.1 µM Minox for 6 h and 12 h. The mRNA level was quantified using TaqMan real-time PCR after treatment.

### 3,4,5-tri-*O*-caffeoylquinic acid (TCQA) upregulated β-catenin expression in human hair follicle dermal papilla cells (HFDPCs) after inhibition with XAV939

XAV939 inhibits the activation of β-catenin stimulating its phosphorylation and non-translocation to the nucleus [33]. Here, HFDPCs were treated with various concentration of XAV939 for 48 h and 10 µM XAV939 was used for further experiment as it didn’t show any cytotoxicity effect on the cells (Figure 5E). After that, the cells were treated with 10 µM XAV939 for 6 and 12 h and results showed that *CTNNB1* expression was significantly decreased upon treatment confirming the inhibitory effect of XAV939 on Wnt/ β-catenin signaling (Figure 5F)**.** The cells were then treated with 10 µM XAV939 (6 and 12 h) and then with 10 µM TCQA (6 and 12 h). Results showed that treatment with TCQA significantly upregulated *CTNNB1* expression suppressing XAV939 inhibition. Finally, after co-treatment with XAV939 and TCQA, the gene expression of β-catenin was maintained higher than after treatment with the inhibitor only. These results further proved the activation of β-catenin by TCQA in DP promoting hair growth cycle.

**Figure 5EF:**
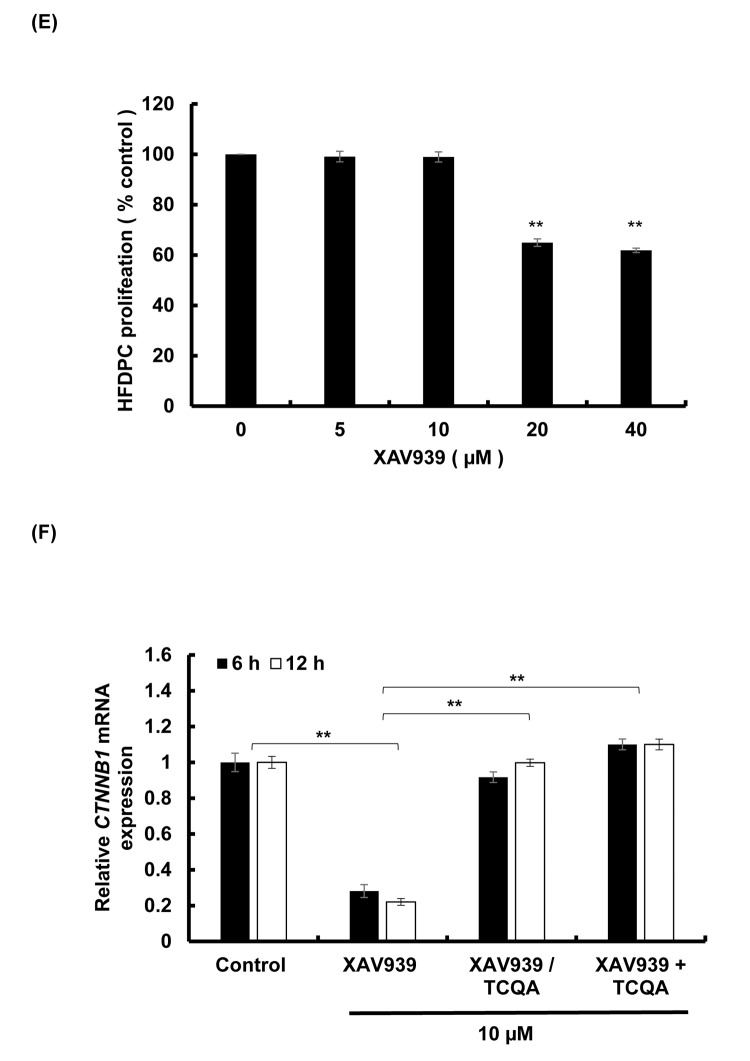
**TCQA stimulated β-catenin expression in human hair follicle dermal papilla cells (HFDPCs)** (**E**) Cell proliferation of HFDPC was assessed after 48 h treatment with various concentrations of XAV939 (β-catenin inhibitor). (**F**) Gene expression expressions of *CTNNB1* (β-catenin) after treatment with 10 µM XAV939 for 6 and 12 h, with 10 µM XAV939 for 6 and 12 h then with 10 µM TCQA for 6 h and 12 h (XAV939/TCQA), and finally with co-treatment of 10 µM XAV939 and 10 µM TCQA for 6 and 12 h (XAV939+TCQA). Results represent the mean ± SD of three independent experiments. *Statistically significant (*P* ≤0.05) difference between control and treated cells. **Statistically significant (*P* ≤0.01) difference between control and treated cells. ##Statistically significant (*P* ≤0.01) difference between Minox-treated cells and TCQA-treated cells.

## DISCUSSION

The hair follicle (HF) goes through a life-long cyclical transformations, progressing through stages of growth, regression, and quiescence, and is considered an established model to study the pathways that govern proliferation, differentiation, and growth [34].

Here, we showed that stimulating Wnt//β-catenin activation with a polyphenolic compound 3,4,5-tri-*O*-caffeoylquinic acid (TCQA) promoted hair growth *in vivo* and *in vitro*. TCQA induced a complete hair recovery in the shaved area of the back of eight-week-old C3H mice (Figure 1A and 1B). The observed results were similar to what have been reported for minoxidil hair regrowth stimulating effect in C3H mice [31]. The HF from TCQA-treated mice displayed a developed morphology compared to control mice (Figure 1C). In addition, during the anagen phase, the inferior segment of the HF is present and grow to form the hair bulb surrounding the dermal papilla (DP), and the hair shaft becomes rooted deep in the sebaceous gland and the dermis [35]. The appearance of the HF from TCQA-treated mice indicates that the hair is in the anagen phase. The anagen phase can be further classified into six sub-stages from anagen I to anagen VI [36]. The regeneration of the HF can be morphologically distinguished starting anagen III and keeps growing until at least anagen VI where the HF reaches its full length, and is entirely surrounded by the IRS, and the bulb and DP reach their optimum size [37]. As shown in Figure 1C, TCQA-treated mice HF are in advanced stage of anagen phase of the hair cycle confirming that TCQA promoted the transition of the telogen phase to the anagen phase.

Several molecular pathways including, Wnt/ β-catenin, Shh, Notch, and FGF are responsible for the maintenance of the HF, the differentiation of the hair matrix cells, and the regulation of the hair growth cycle [38,39]. An upregulation of genes involved in Wnt/β-catenin signaling, β-catenin binding, and transcription, and a downregulation in genes relevant in glycosylation, protein binding, and β-catenin degradation complex was observed in TCQA-treated group (Figure 2).

TCQA upregulated Wnt/β-catenin-related genes*,* *Wnt2b, Wls, Wnt4,* and *Ctnnb1* (Table 1). β-catenin targets *Foxn1* involved in the activation of the matrix cells that differentiated to form the hair shaft to regenerate the HF [40–42]. The HFs of TCQA-treated mice were in the anagen phase and this was clearly caused by increased Wnt proteins level known to initiate HF development, and in addition, by the upregulation of β-catenin and its target *Foxn1.* Various studies have shown that β-catenin is strongly expressed during anagen phase in the DP and in the outer root sheath, and the absence of β-catenin induces a premature catagen phase [40,43,44]. In this study, TCQA caused β-catenin to move to the DP, upregulated its expression in mice treated skin confirming that the HF is in the anagen phase (Figure 3). Fibroblast growth factors (FGF) pathway-related genes *Fgf1* and *Fgf2* are required, respectively, in the promotion of the anagen phase in telogenic C57BL/6 mice, and the stimulation of hair growth [38,45]. In Table 1, we observed an upregulation of these genes. The increase in FGF-related genes expression may be attributed to the stimulation of Wnt/β-catenin as the absence of Wnt/β-catenin signaling alters FGF signaling genes [32]. Microarray results also revealed an upregulation in the expression of Notch signaling-associated genes including, *RBPj* and *Tspan10. RBPj* binds to Notch in the nucleus to trans-activate the transcription of hair growth-associated genes*,* and the mutation of which is associated with hair loss and impaired matrix cell differentiation, and *Tspan10* is involved in the regulation of hair growth [46–49]. More specifically, the expression of several transcription factors regulated by Notch such as *Dlx3* was enhanced. Mutation in *Dlx3* causes alopecia due to hair shaft differentiation failure [17,50]. On the other hand, an upregulation in DP marker was observed including *Alpl* and Corin (Table 1). Alkaline phosphatase (ALP) is known to be marker of DP cells expressed through the hair cycle, as for Corin is reported to be upregulated in DP during anagen phase [10]. A summary of TCQA-modulated genes linked to cell cycle, ATP binding, and oxidation-reduction process is presented in Table 2, and one of these genes is *Smchd1* with an ATP binding function (Table 2). During the changes of hair cycle from telogen to anagen phase in guinea-pig, ATP content increase following by a development of the size of the HF and an expansion in DNA content [51].

Other studies have described the role of Wnt/β-catenin and its interaction with AKT and ERK pathways to promote hair growth [31,52]. In this paper, an upregulation of Wnt/β-catenin and its interaction with other pathways that are linked to hair growth was observed.

Rac/Ras-related genes are involved in cell migration, homeostatic, structural plasticity, and memory [53–55]. It has been reported that Rac/Ras pathway is expressed in the inner ear and may affect hair cell development, and Rho and Rac protein are activated by Wnt/ β-catenin signaling and this activation is required for vertebrate gastrulation [18,56–58]. TCQA modulated Rac/Ras- genes, *Rasgrp,* *Dock2, Rassf1,* *Dgki,* and *Plk2* (Table 2)*.* The effect of Wnt/β-catenin on Rac/Ras pathway regulation and involvement in hair growth stimulation has not yet been assessed, but here, we report for the first time that TCQA can, through Wnt/ β-catenin signaling activates Rac/Ras pathway, and as a result promoted hair development.

TCQA downregulated genes repressing Wnt/β-catenin pathway including, *Gsk3b, Tcf3, Igfbp4,* and *Shisa3* (Table 3). Gsk3b phosphorylates β-catenin leading to its degradation and non-translocation to the nucleus inducing catagen phase in the HF [59]. Tcf3 and Igfbp4 inhibit Wnt/ β-catenin signaling during neocortical development [60,61]. Other genes were negatively modulated by TCQA like *Aebp1* and *Nfatc1*. *Aebp1* is only expressed in the HF at telogen phase and it is known to regulate MAPK pathway [62]. *Nfatc1* regulates HF stem cells quiescence, and is upregulated during telogen, and its expression level decreases during the transition from telogen to anagen in order to reduce the quiescence of stem cells promoting the proliferation [63–65]. A downregulation in genes upregulated with aging such as *Egr1* and *Fos* was observed (Table 4). Here, we introduce TCQA as drug to accelerate hair cycle and prevent hair aging and loss.

The HF goes through a continual cycle regulated by the DP that maintains the stem cells niche during telogen and its activates them during anagen [7,65,66]. As mentioned earlier in this paper, the ATP content increases during anagen promoting the expansion of the size of DP [51]. TCQA not only stimulated the proliferation of human hair follicle dermal papilla cells (HFDPCs) but also the ATP content (Figure 4A and 4C) and this is correlated with the upregulation of *Smchd1 in vivo* (Table 2). In the DP, β-catenin activity is strongly associated with the anagen phase prolongation, and it direct the hair shaft morphogenesis while regulating the expression of secreted growth factors [44]. After treatment with TCQA, the gene and the protein expression of β-catenin was strongly enhanced in the DP and this explains the fast entry of TCQA-treated mice HFs into the anagen phase (Figures 1 and 5).

On the other hand, a knockdown of Tankyrase known to play a role in the activation of Wnt/ β-catenin leads to tumorigenesis [67]. XAV939 is a small molecule that inhibit Tankyrase inducing β-catenin phosphorylation and non-activation in lung adenocarcinoma A549 cell [68]. The inhibitory effect of XAV939 on β-catenin in DP cells has not yet been checked. In this study, XAV939 downregulated β-catenin gene expression (*CTNNB1*) in HFDPCs, however, after treatment with TCQA, *CTNNB1* expression significantly upregulated again (Figure 5F). Moreover, after a co-treatment of XAV939 and TCQA, *CTNNB1* expression remained unchanged proving that TCQA target specifically β-catenin and inhibit its phosphorylation.

In summary, the activation of β-catenin stimulated the expression of Wnt proteins, Rac/Ras pathway, and hair growth-associated genes include FGF- and Notch-related genes promoting the transition from telogen to anagen, anagen initiation and elongation, hair matrix differentiation, and hair shaft development (Figure 3E).

Furthermore, the observed stimulation of hair growth cycle was supported by the downregulation of telogen- and aging-associated genes that caused bulge stem cells to migrate and differentiate promoting the anagen induction. The repression of Wnt signal inhibitors contributed to further enhance the effect of β-catenin and Wnt proteins activation.

Clinical studies would be however necessary to validate the observed hair growth promotion effect of TCQA. The potential effect of the activation of β-catenin by TCQA on hair pigmentation and follicular melanocytes will be looked at as β-catenin regulates the transcription factor of the melanogenesis enzyme microphthalmia-associated transcription factor (MITF).

## MATERIALS AND METHODS

### Sample preparation

Synthesized 3,4,5-tri-*O*-caffeoylquinic acid (TCQA) with 97% purity was provided by Dr. Kozo Sato from Synthetic Organic Chemistry Laboratories, the FUJIFILM Corporation (Kanagawa, Japan). For the *in vivo* experiment, TCQA was prepared by first dissolving in a small volume of 70% ethanol and then diluted in purified water. For the *in vitro* assay, a highly concentration of TCQA was dissolved in 70% ethanol and then diluted in cell culture medium.

### Animal experiment

Eight-weeks-old male C3H mice were purchased from Charles River Laboratories, Japan Inc. (Kanagawa, Japan) and housed individually in cages at Gene Research Center of the University of Tsukuba. After one week of acclimatization, the mice were randomly assigned to experimental groups (n=5 for each group) TCQA-treated group and milli-Q water treated group (control group). The mice were anesthetized using isoflurane (Wako Pure Chemical Industries, Tokyo, Japan), then the telogen hair on the dorsal part of the mice back were shaved using hair clipper in order to induce the anagen phase. Each mouse then received a topical application of 1% TCQA or water daily for four weeks. The dorsal part of the shaved area was observed and photographed daily starting on the 14^th^ day after treatment. At the end of the treatment period, the mice were sacrificed by cervical spine dislocation and the skin collected from the treated area were divided into four parts and washed with phosphate-buffered saline (PBS), then immediately immersed in liquid nitrogen and kept at −80 °C. The ImageJ processing program (National Institutes of Health, Bethesda, USA) was used to measure the hair regrowth in the treated area. The experiment was approved by the Animal Study Committee of the University of Tsukuba (No.17-060) and the procedures were handled according to the guidelines of the Care and Use and Use of Animals approved by the Council of the Physiological Society of Japan.

### DNA microarray

RNA used as a template were extracted from skin tissues collected from treated area of mice dorsal part using ISOGEN solution (Nippon Gene, Tokyo, Japan) following the manufacturer’s instructions. The total RNA extracted were then amplified and labeled. The labeled fragmented aRNA was then hybridized to the Affymetrix mouse Array strips (Affymetrix, Santa Clara, USA). The GeneChip (Mouse Genome 430 2.0 Array) was washed, stained, and scanned using Affymetrix GeneAtlas Imaging Station to obtain the mRNA expression of various genes from mouse genome. The gene ontology, biological process, and fold-change in gene expression (2-fold change, control vs TCQA) were then analyzed using Transcriptome Analysis Console (TAC) Software (version 4.0.1) and database for annotation, visualization, and integrated discovery (DAVID) bioinformatics resources 6.8 [69,70]. The average signal log2 (control vs TCQA) was subjected to hierarchical clustering using Euclidean distance and average linkage algorithm of the TIGR Mev version 3.0.3 software (The Institute for Genomic Research, MD, USA).

### Immunohistochemistry

Skin tissues from mice treated with TCQA and milli-Q water were collected, embedded in optimum cutting temperature (OCT) compound, and then cut at a thickness of 10 µM. The sections were then dried and fixed in 4% paraformaldehyde (SIGMA, Saint Louis, USA) and washed in different solutions of PBS, 20 mM glycine/PBS (SIGMA, Saint Louis, USA), and 0.1% v/v Triton X-100 /PBS (SIGMA, Saint Louis, USA). The skin samples were then blocked and immersed in a solution of 1:100 dilution of rabbit anti- β-catenin (Abcam, Rockford, USA) for overnight at 4°C in a wet chamber. Then washing in 0.1% v/v Triton X-100 /PBS was conducted. The samples were incubated with Alexa 594-conjugated anti-rabbit (Abcam, Rockford, USA) for 1 h at room temperature (RT) and stained with Hoechst and mounted in antifade solution (*p*-phenylenediamine, PBS, and glycerol).

### Cells and cell culture

Human hair follicle dermal papilla cells (HFDPCs) were purchased from Cell Application Inc. (Tokyo, Japan). HFDPCs were maintained in papilla cell growth medium (Toyobo, Osaka, Japan) supplemented with growth factors: fetal calf serum, insulin transferrin triiodothyronine, bovine pituitary extract, and cyproterone solution (Toyobo, Osaka, Japan).

Human epidermal melanocytes (HEM) were purchased from Gibco Invitrogen cell culture. HEM were maintained in Medium 254 (Gibco, South America) supplemented with human melanocyte growth supplement HMGS (Gibco). The medium was changed every other day until the cells were 60% confluent and ready to be subcultured.

The cells were kept under sterile conditions at 37 °C in 75-cm^2^ flask (BD Falcon, England, UK) in a humidified atmosphere of 5% CO_2_. The viability of the cells was determined using trypan blue exclusion method.

### Cell proliferation assay

The effect of TCQA on cell proliferation was assessed using the 3-(4,5-dimethyl-thiazol-2-y1) 2,5-diphenyl tetrazolium bromide or MTT assay (Dojindo, Kumamoto, Japan). The cells were seeded in 96-well plate at 3×10^5^ cells/well at 37 °C. After 24 h seeding, the growth medium was replaced by various concentrations of TCQA and incubated for 48 h and 72 h. MTT reagent (5 mg/ml) was added to the cells and incubated further for 8 h followed by an addition of 10% sodium dodecyl sulfate (SDS) and incubation overnight. Absorbance was measured at 570 nm using microplate reader (Powerscan HT, NJ, USA). The viability of the cells was quantiﬁed as the percentage (%) of living cells relative to the control (untreated cells). To detect any change in the morphology, the cells were observed using a contrast microscope (Leica Microsystems, Wetzlar, Germany).

### Adenosine triphosphate (ATP) assay

Luminescence luciferase assay kit (Toyo Ink, Tokyo, Japan) was used to measure the ATP content. HFDPCs were seeded in 96-well plate at 3×10^5^ cells/ 100 µl well for 24 h, then the medium was replaced by fresh culture medium containing various concentrations of TCQA or 0.1 µM of the positive control minoxidil (Tokyo Chemical Industry, Tokyo, Japan). After 24 h and 48 h, the plate was incubated for 15 min at RT, then 100 µl of ATP reagent was added, followed by homogenization, and 1 min incubation in the dark. Then, 150 µl of the suspension was transferred to white 96-well plate and incubated for 10 min at RT. Intracellular ATP content was measured by luminescence and calculated as the percentage (%) of TCQA treated-cells relative to the control (untreated cells).

### RNA extraction

HFDPCs were seeded at a density of 5×10^5^ cells per 100-mm petri dish. The cells were allowed to attach overnight and then the growth medium was replaced with a fresh one containing 0 and 10 µM of TCQA or 0.1 µM minoxidil used as positive control (Tokyo Chemical Industry, Tokyo, Japan). HFDPCs were treated as well with only 10 µM XAV939 (SIGMA, Saint Louis, USA), then with 10 µM XAV939 following by a further incubation with 10 µM TCQA (XAV939/TCQA), and finally co-treated with 10 µM XAV939 and TCQA simultaneously (XAV939+TCQA). After 6 and 12 h treatment, the growth medium was removed, and the cells washed with cold PBS before the total RNA was extracted using ISOGEN kit (Nippon Gene, Tokyo, Japan) following the manufacturer’s instructions. The RNA concentration was assessed using a NanoDrop 2000 spectrophotometer (NanoDrop Technologies, Massachusetts, USA).

### Quantitative real-time PCR analysis

The extracted RNAs were used as templates for reverse transcription PCR using SuperScript III reverse transcription kit (Invitrogen, CA, USA). The cycling protocol is as follows: 95 °C for 10 min, 40 cycles of 95 °C for 15 s, and 60 °C for 1 min. TaqMan Universal PCR mix and TaqMan probes specific to *Ctnnb1* (Mm 00483039_ m1), *CTNNB1* (Hs99999168_m1), and *ALPL* (Hs01029144) (Applied Biosystems, CA, USA) were used for real-time PCR. The real-Time PCR was performed using 7500 Fast Real-Time PCR Software 1.3.1 (Applied Biosystems, CA, USA)*. Gapdh* (Mm99999915_g1) and *GAPDH* (Hs 02786624_ g1) (Applied Biosystems, CA, USA) were used as endogenous control. All reactions were run in triplicates.

### Protein extraction

HFDPCs were seeded at density of 5×10^5^ cells per 100-mm petri dish. After overnight incubation, the medium was removed and the cells were treated with 10 µM TCQA and 0.1 µM minoxidil (Tokyo Chemical Industry, Tokyo, Japan). After 12 h and 24 h treatment, total protein extraction was achieved using radio-immunoprecipitation assay (RIPA) buffer (SIGMA, Saint Louis, USA) and protease inhibitor following the manufacturer’s instructions.

Proteins from tissues collected from treated area at mice dorsal part were also extracted. The skin sections were crushed using a homogenizer in RIPA buffer (SIGMA, Saint Louis, USA) and protease inhibitor following the manufacturer’s instructions.

Protein samples (15 µg) were quantified using 2-D Quant kit according to manufacturer’s instructions (GE Healthcare, Chicago, USA).

### Western blot

The protein samples were resolved in 10% sodium dodecyl sulphate-polyacrylamide gel electrophoresis (SDS-PAGE) and transferred to polyvinylidene difluoride membrane (PVDF) (Millipore, NJ, USA). The proteins were blotted with β-catenin 71-2700 (Thermo Fisher Scientific, Massachusetts, USA) and GAPDH sc32233 (Santa Cruz Biotechnology, Texas, USA) primary antibodies and incubated with the second antibody goat anti-rabbit IRDye 800 CW or IRDye 680 LT goat anti-mouse and the expression detected using LI-COR Odyssey Infrared Imaging System (LI-COR, NE, USA).

### Immunocytochemistry

The cells were seeded at density of 3×10^4^/well in lab-tek slides chambers (SIGMA, Saint Louis, USA), and then allowed to attached for overnight at 37 °C. HFDPCs were treated with 0, and 10 µM TCQA and 0.1 µM Minox for 24 h. The medium was then removed and the cells were washed with 0.1% v/v Triton X-100 /PBS (SIGMA, Saint Louis, USA). After 1 h incubation at RT with the blocking solution, the first antibody rabbit anti- β-catenin (Abcam, Rockford, USA) was added for overnight at 4 °C. Therefore, the cells were immersed in a solution of 1:10000 dilution of Alexa 594-conjugated anti-rabbit (Abcam, Rockford, USA) and mounted with DAPI.

### Statistical analysis

Results were expressed as mean ± standard deviation (SD). Statistical analysis was performed using Student’s t-test when comparing two value sets (control vs TCQA). *P* value of ≤0.05 was considered significant. ANOVA (One-way between-subject ANOVA unpaired) was performed to assess the level of significance between treated groups *in* vivo. The gene in gene expression was considered significant when change in expression was at least 2-fold (control vs TCQA). ANOVA (one way between-subject ANOVA unpaired) was performed to assess the level of significance between Minox and TCQA-treated cells; A *P* value of ≤0.05 was considered significant.

## References

[1] Rushton DH, Norris MJ, Dover R, Busuttil N. Causes of hair loss and the developments in hair rejuvenation. Int J Cosmet Sci. 2002; 24:17–23. 10.1046/j.0412-5463.2001.00110.x 18498491

[2] Patel M, Harrison S, Sinclair R. Drugs and hair loss derm clinics. Dermatol Clin. 2013; 31:3065 10.1016/j.det.2012.08.002 23159177

[3] Rosenquist TA, Martin GR. Fibroblast growth factor signalling in the hair growth cycle: expression of the fibroblast growth factor receptor and ligand genes in the murine hair follicle. Dev Dyn. 1996; 205:379–86. 10.1002/(SICI)1097-0177(199604)205:4<379::AID-AJA2>3.0.CO;2-F 8901049

[4] Paus R, Cotsarelis G, He B. The biology of hair follicles. N Engl J Med. 1999; 341:491–97. 10.1056/NEJM199908123410706 10441606

[5] Cotsarelis G, Sun TT, Lavker RM. Label-retaining cells reside in the bulge area of pilosebaceous unit: implications for follicular stem cells, hair cycle, and skin carcinogenesis. Cell. 1990; 61:1329–37. 10.1016/0092-8674(90)90696-C 2364430

[6] Sano S, Kira M, Takagi S, Yoshikawa K, Takeda J, Itami S. Correction for Sano et al. Two distinct signaling pathways in hair cycle induction: Stat3-dependent and -independent pathways. Proc Natl Acad Sci USA. 2015; 112:201506638. 10.1073/pnas.240303097 11087819 PMC17660

[7] Jahoda CA, Horne KA, Oliver RF. Induction of hair growth by implantation of cultured dermal papilla cells. Nature. 1984; 311:560–62. 10.1038/311560a0 6482967

[8] Elliott K, Stephenson TJ, Messenger AG. Differences in hair follicle dermal papilla volume are due to extracellular matrix volume and cell number: implications for the control of hair follicle size and androgen responses. J Invest Dermatol. 1999; 113:873–77. 10.1046/j.1523-1747.1999.00797.x 10594724

[9] Millar SE, Willert K, Salinas PC, Roelink H, Nusse R, Sussman DJ, Barsh GS. WNT signaling in the control of hair growth and structure. Dev Biol. 1999; 207:133–49. 10.1006/dbio.1998.9140 10049570

[10] Driskell RR, Clavel C, Rendl M, Watt FM. Hair follicle dermal papilla cells at a glance. J Cell Sci. 2011; 124:1179–82. 10.1242/jcs.082446 21444748 PMC3115771

[11] Messenger AG. The control of hair growth: an overview. J Invest Dermatol. 1993 (Suppl ); 101:4S–9S. 10.1016/0022-202X(93)90494-3 8326154

[12] Kishimoto J, Burgeson RE, Morgan BA. Wnt signaling maintains the hair-inducing activity of the dermal papilla. Genes Dev. 2000; 14:1181–85. 10817753 PMC316619

[13] Visweswaran M, Pohl S, Arfuso F, Newsholme P, Dilley R, Pervaiz S, Dharmarajan A. Multi-lineage differentiation of mesenchymal stem cells - To Wnt, or not Wnt. Int J Biochem Cell Biol. 2015; 68:139–47. 10.1016/j.biocel.2015.09.008 26410622

[14] Choi YS, Zhang Y, Xu M, Yang Y, Ito M, Peng T, Cui Z, Nagy A, Hadjantonakis AK, Lang RA, Cotsarelis G, Andl T, Morrisey EE, Millar SE. Distinct functions for Wnt/β-catenin in hair follicle stem cell proliferation and survival and interfollicular epidermal homeostasis. Cell Stem Cell. 2013; 13:720–33. 10.1016/j.stem.2013.10.003 24315444 PMC3900235

[15] McNeill H, Woodgett JR. When pathways collide: collaboration and connivance among signalling proteins in development. Nat Rev Mol Cell Biol. 2010; 11:404–13. 10.1038/nrm2902 20461097 PMC4489880

[16] Guo L, Yu QC, Fuchs E. Targeting expression of keratinocyte growth factor to keratinocytes elicits striking changes in epithelial differentiation in transgenic mice. EMBO J. 1993; 12:973–86. 10.1002/j.1460-2075.1993.tb05738.x 7681397 PMC413298

[17] Pan Y, Lin MH, Tian X, Cheng HT, Gridley T, Shen J, Kopan R. γ-secretase functions through Notch signaling to maintain skin appendages but is not required for their patterning or initial morphogenesis. Dev Cell. 2004; 7:731–43. 10.1016/j.devcel.2004.09.014 15525534

[18] Habas R, Dawid IB, He X. Coactivation of Rac and Rho by Wnt/Frizzled signaling is required for vertebrate gastrulation. Genes Dev. 2003; 17:295–309. 10.1101/gad.1022203 12533515 PMC195976

[19] Huelsken J, Vogel R, Erdmann B, Cotsarelis G, Birchmeier W. β-Catenin controls hair follicle morphogenesis and stem cell differentiation in the skin. Cell. 2001; 105:533–45. 10.1016/S0092-8674(01)00336-1 11371349

[20] Ouji Y, Yoshikawa M, Moriya K, Nishiofuku M, Matsuda R, Ishizaka S. Wnt-10b, uniquely among Wnts, promotes epithelial differentiation and shaft growth. Biochem Biophys Res Commun. 2008; 367:299–304. 10.1016/j.bbrc.2007.12.091 18155657

[21] Zhou L, Xu M, Yang Y, Yang K, Wickett RR, Andl T, Millar SE, Zhang Y. Activation of β-Catenin Signaling in CD133-Positive Dermal Papilla Cells Drives Postnatal Hair Growth. PLoS One. 2016; 11:e0160425. 10.1371/journal.pone.0160425 27472062 PMC4966972

[22] Van Neste D, Fuh V, Sanchez-Pedreno P, Lopez-Bran E, Wolff H, Whiting D, Roberts J, Kopera D, Stene JJ, Calvieri S, Tosti A, Prens E, Guarrera M, et al. Finasteride increases anagen hair in men with androgenetic alopecia. Br J Dermatol. 2000; 143:804–10. 10.1046/j.1365-2133.2000.03780.x 11069460

[23] Kwack MH, Kang BM, Kim MK, Kim JC, Sung YK. Minoxidil activates β-catenin pathway in human dermal papilla cells: a possible explanation for its anagen prolongation effect. J Dermatol Sci. 2011; 62:154–59. 10.1016/j.jdermsci.2011.01.013 21524889

[24] Rathi V, Rathi JC, Tamizharasi S, Kumar A. Phcog Rev.: Short Review Plants used for hair growth promotion : A review. Rev Am Soc. 2008; 2:185–87.

[25] Miyamae Y, Han J, Sasaki K, Terakawa M, Isoda H, Shigemori H. 3,4,5-tri-O-caffeoylquinic acid inhibits amyloid β-mediated cellular toxicity on SH-SY5Y cells through the upregulation of PGAM1 and G3PDH. Cytotechnology. 2011; 63:191–200. 10.1007/s10616-011-9341-1 21424281 PMC3080471

[26] Han J, Miyamae Y, Shigemori H, Isoda H. Neuroprotective effect of 3,5-di-O-caffeoylquinic acid on SH-SY5Y cells and senescence-accelerated-prone mice 8 through the up-regulation of phosphoglycerate kinase-1. Neuroscience. 2010; 169:1039–45. 10.1016/j.neuroscience.2010.05.049 20570715

[27] Kimura Y, Okuda H, Okuda T, Hatano T, Agata I, Arichi S. Studies on the activities of tannins and related compounds from medicinal plants and drugs. VI. Inhibitory effects of caffeoylquinic acids on histamine release from rat peritoneal mast cells. Chem Pharm Bull (Tokyo). 1985; 33:690–96. 10.1248/cpb.33.690 2410155

[28] Kim HJ, Kim JS, Woo JT, Lee IS, Cha BY. Hyperpigmentation mechanism of methyl 3,5-di-caffeoylquinate through activation of p38 and MITF induction of tyrosinase. Acta Biochim Biophys Sin (Shanghai). 2015; 47:548–56. 10.1093/abbs/gmv040 26018825

[29] Tang B, Huang Y, Yang H, Tang P, Li H. Molecular mechanism of the binding of 3,4,5-tri-O-caffeoylquinic acid to human serum albumin: saturation transfer difference NMR, multi-spectroscopy, and docking studies. J Photochem Photobiol B. 2016; 165:24–33. 10.1016/j.jphotobiol.2016.10.017 27768950

[30] Sasaki K, Davies J, Doldán NG, Arao S, Ferdousi F, Szele FG, Isoda H. 3,4,5-Tricaffeoylquinic acid induces adult neurogenesis and improves deficit of learning and memory in aging model senescence-accelerated prone 8 mice. Aging (Albany NY). 2019; 11:401–22. . t 10.18632/aging.101748 30654329 PMC6366991

[31] Lee SH, Yoon J, Shin SH, Zahoor M, Kim HJ, Park PJ, Park WS, Min S, Kim HY, Choi KY. Valproic acid induces hair regeneration in murine model and activates alkaline phosphatase activity in human dermal papilla cells. PLoS One. 2012; 7:e34152. 10.1371/journal.pone.0034152 22506014 PMC3323655

[32] Tsai SY, Sennett R, Rezza A, Clavel C, Grisanti L, Zemla R, Najam S, Rendl M. Wnt/β-catenin signaling in dermal condensates is required for hair follicle formation. Dev Biol. 2014; 385:179–88. 10.1016/j.ydbio.2013.11.023 24309208 PMC3933391

[33] Kulak O, Chen H, Holohan B, Wu X, He H, Borek D, Otwinowski Z, Yamaguchi K, Garofalo LA, Ma Z, Wright W, Chen C, Shay JW, et al. Disruption of Wnt/β-Catenin Signaling and Telomeric Shortening Are Inextricable Consequences of Tankyrase Inhibition in Human Cells. Mol Cell Biol. 2015; 35:2425–35. 10.1128/MCB.00392-15 25939383 PMC4475917

[34] Millar SE. Molecular mechanisms regulating hair follicle development. J Invest Dermatol. 2002; 118:216–25. 10.1046/j.0022-202x.2001.01670.x 11841536

[35] Sinclair R, Jolley D, Mallari R, Magee J, Tosti A, Piracinni BM, Vincenzi C, Happle R, Ferrando J, Grimalt R, Thérèse L, Van Neste D, Zlotogorski A, et al. Morphological approach to hair disorders. J Investig Dermatol Symp Proc. 2003; 8:56–64. 10.1046/j.1523-1747.2003.12172.x 12894995

[36] Kobielak K, Pasolli HA, Alonso L, Polak L, Fuchs E. Defining BMP functions in the hair follicle by conditional ablation of BMP receptor IA. J Cell Biol. 2003; 163:609–23. 10.1083/jcb.200309042 14610062 PMC2173651

[37] Müller-Röver S, Handjiski B, van der Veen C, Eichmüller S, Foitzik K, McKay IA, Stenn KS, Paus R. A comprehensive guide for the accurate classification of murine hair follicles in distinct hair cycle stages. J Invest Dermatol. 2001; 117:3–15. 10.1046/j.0022-202x.2001.01377.x 11442744

[38] Lin WH, Xiang LJ, Shi HX, Zhang J, Jiang LP, Cai PT, Lin ZL, Lin BB, Huang Y, Zhang HL, Fu XB, Guo DJ, Li XK, et al. Fibroblast growth factors stimulate hair growth through β-catenin and Shh expression in C57BL/6 mice. BioMed Res Int. 2015; 2015:730139. 10.1155/2015/730139 25685806 PMC4313060

[39] Lee J, Tumbar T. Hairy tale of signaling in hair follicle development and cycling. Semin Cell Dev Biol. 2012; 23:906–16. 10.1016/j.semcdb.2012.08.003 22939761 PMC3496046

[40] Andl T, Reddy ST, Gaddapara T, Millar SE. WNT signals are required for the initiation of hair follicle development. Dev Cell. 2002; 2:643–53. 10.1016/S1534-5807(02)00167-3 12015971

[41] Lowry WE, Blanpain C, Nowak JA, Guasch G, Lewis L, Fuchs E. Defining the impact of β-catenin/Tcf transactivation on epithelial stem cells. Genes Dev. 2005; 19:1596–611. 10.1101/gad.1324905 15961525 PMC1172065

[42] Li J, Baxter RM, Weiner L, Goetinck PF, Calautti E, Brissette JL. Foxn1 promotes keratinocyte differentiation by regulating the activity of protein kinase C. Differentiation. 2007; 75:694–701. 10.1111/j.1432-0436.2007.00176.x 17459087

[43] Maretto S, Cordenonsi M, Dupont S, Braghetta P, Broccoli V, Hassan AB, Volpin D, Bressan GM, Piccolo S. Mapping Wnt/beta-catenin signaling during mouse development and in colorectal tumors. Proc Natl Acad Sci USA. 2003; 100:3299–304. 10.1073/pnas.0434590100 12626757 PMC152286

[44] Enshell-Seijffers D, Lindon C, Kashiwagi M, Morgan BA. β-catenin activity in the dermal papilla regulates morphogenesis and regeneration of hair. Dev Cell. 2010; 18:633–42. 10.1016/j.devcel.2010.01.016 20412777 PMC2893731

[45] Takabayashi Y, Nambu M, Ishihara M, Kuwabara M, Fukuda K, Nakamura S, Hattori H, Kiyosawa T. Enhanced effect of fibroblast growth factor-2-containing dalteparin/protamine nanoparticles on hair growth. Clin Cosmet Investig Dermatol. 2016; 9:127–34. 10.2147/CCID.S108187 27274299 PMC4876681

[46] Yamamoto N, Tanigaki K, Han H, Hiai H, Honjo T. Notch/RBP-J signaling regulates epidermis/hair fate determination of hair follicular stem cells. Curr Biol. 2003; 13:333–38. 10.1016/S0960-9822(03)00081-2 12593800

[47] Blanpain C, Lowry WE, Pasolli HA, Fuchs E. Canonical notch signaling functions as a commitment switch in the epidermal lineage. Genes Dev. 2006; 20:3022–35. 10.1101/gad.1477606 17079689 PMC1620020

[48] Honjo T. The shortest path from the surface to the nucleus: RBP-J κ/Su(H) transcription factor. Genes Cells. 1996; 1:1–9. 10.1046/j.1365-2443.1996.10010.x 9078362

[49] Dornier E, Coumailleau F, Ottavi JF, Moretti J, Boucheix C, Mauduit P, Schweisguth F, Rubinstein E. TspanC8 tetraspanins regulate ADAM10/Kuzbanian trafficking and promote Notch activation in flies and mammals. J Cell Biol. 2012; 199:481–96. 10.1083/jcb.201201133 23091066 PMC3483123

[50] Demehri S, Kopan R. Notch signaling in bulge stem cells is not required for selection of hair follicle fate. Development. 2009; 136:891–96. 10.1242/dev.030700 19211676 PMC2727555

[51] Adachi K, Watanabe Y, Inouye K. Activity of glucose-6-phosphate 1-dehydrogenase in hair follicles with male-pattern alopecia. Biosci Biotechnol Biochem. 1999; 63:2219–21. 10.1271/bbb.63.2219 10664855

[52] Kang JI, Kim MK, Lee JH, Jeon YJ, Hwang EK, Koh YS, Hyun JW, Kwon SY, Yoo ES, Kang HK. Undariopsis peterseniana promotes hair growth by the activation of Wnt/β-catenin and ERK pathways. Mar Drugs. 2017; 15. 10.3390/md15050130 28475144 PMC5450536

[53] Stone JC. Regulation and function of the rasGRP family of ras activators in blood cells. Genes Cancer. 2011; 2:320–34. 10.1177/1947601911408082 21779502 PMC3128638

[54] Brugnera E, Haney L, Grimsley C, Lu M, Walk SF, Tosello-Trampont AC, Macara IG, Madhani H, Fink GR, Ravichandran KS. Unconventional Rac-GEF activity is mediated through the Dock180-ELMO complex. Nat Cell Biol. 2002; 4:574–82. 10.1038/ncb824 12134158

[55] Lee KJ, Lee Y, Rozeboom A, Lee JY, Udagawa N, Hoe HS, Pak DT. Requirement for Plk2 in orchestrated ras and rap signaling, homeostatic structural plasticity, and memory. Neuron. 2011; 69:957–73. 10.1016/j.neuron.2011.02.004 21382555 PMC3073828

[56] Habas R, He X. Activation of Rho and Rac by Wnt/frizzled signaling. Methods Enzymol. 2006; 406:500–11. 10.1016/S0076-6879(06)06038-1 16472682

[57] Kollmar R. Who does the hair cell’s 'do? Rho GTPases and hair-bundle morphogenesis. Curr Opin Neurobiol. 1999; 9:394–98. 10.1016/S0959-4388(99)80059-2 10448167

[58] Kalinec F, Zhang M, Urrutia R, Kalinec G. Rho GTPases mediate the regulation of cochlear outer hair cell motility by acetylcholine. J Biol Chem. 2000; 275:28000–05. 10862776 10.1074/jbc.M004917200

[59] Wu G, Huang H, Garcia Abreu J, He X. Inhibition of GSK3 phosphorylation of β-catenin via phosphorylated PPPSPXS motifs of Wnt coreceptor LRP6. PLoS One. 2009; 4:e4926. 10.1371/journal.pone.0004926 19293931 PMC2654145

[60] Kuwahara A, Sakai H, Xu Y, Itoh Y, Hirabayashi Y, Gotoh Y. Tcf3 represses Wnt-β-catenin signaling and maintains neural stem cell population during neocortical development. PLoS One. 2014; 9:e94408. 10.1371/journal.pone.0094408 24832538 PMC4022625

[61] Zhu W, Shiojima I, Ito Y, Li Z, Ikeda H, Yoshida M, Naito AT, Nishi J, Ueno H, Umezawa A, Minamino T, Nagai T, Kikuchi A, et al. IGFBP-4 is an inhibitor of canonical Wnt signalling required for cardiogenesis. Nature. 2008; 454:345–49. 10.1038/nature07027 18528331

[62] Geyfman M, Gordon W, Paus R, Andersen B. Identification of telogen markers underscores that telogen is far from a quiescent hair cycle phase. J Invest Dermatol. 2012; 132:721–24. 10.1038/jid.2011.367 22089832 PMC3278528

[63] Horsley V, Aliprantis AO, Polak L, Glimcher LH, Fuchs E. NFATc1 balances quiescence and proliferation of skin stem cells. Cell. 2008; 132:299–310. 10.1016/j.cell.2007.11.047 18243104 PMC2546702

[64] Keyes BE, Segal JP, Heller E, Lien WH, Chang CY, Guo X, Oristian DS, Zheng D, Fuchs E. Nfatc1 orchestrates aging in hair follicle stem cells. Proc Natl Acad Sci USA. 2013; 110:E4950–59. 10.1073/pnas.1320301110 24282298 PMC3870727

[65] Avigad Laron E, Aamar E, Enshell-Seijffers D. The Mesenchymal Niche of the Hair Follicle Induces Regeneration by Releasing Primed Progenitors from Inhibitory Effects of Quiescent Stem Cells. Cell Reports. 2018; 24:909–921.e3. 10.1016/j.celrep.2018.06.084 30044987

[66] Fuchs E, Merrill BJ, Jamora C, DasGupta R. At the roots of a never-ending cycle. Dev Cell. 2001; 1:13–25. 10.1016/S1534-5807(01)00022-3 11703920

[67] McGonigle S, Chen Z, Wu J, Chang P, Kolber-Simonds D, Ackermann K, Twine NC, Shie JL, Miu JT, Huang KC, Moniz GA, Nomoto K. E7449: A dual inhibitor of PARP1/2 and tankyrase1/2 inhibits growth of DNA repair deficient tumors and antagonizes Wnt signaling. Oncotarget. 2015; 6:41307–23. 10.18632/oncotarget.5846 26513298 PMC4747407

[68] Li C, Zheng X, Han Y, Lv Y, Lan F, Zhao J. XAV939 inhibits the proliferation and migration of lung adenocarcinoma A549 cells through the WNT pathway. Oncol Lett. 2018; 15:8973–82. 29805633 10.3892/ol.2018.8491 PMC5958670

[69] Huang W, Sherman BT, Lempicki RA. Bioinformatics enrichment tools: paths toward the comprehensive functional analysis of large gene lists. Nucleic Acids Res. 2009; 37:1–13. 10.1093/nar/gkn923 19033363 PMC2615629

[70] Zacariotti RL, do Valle R R. Observation of mating in the Calico Snake Oxyrhopus petola Linnaeus, 1758. Herpetol Notes. 2010; 3:139–40.

